# Risk of COVID-19 infection among prison staff in the United States

**DOI:** 10.1186/s12889-021-11077-0

**Published:** 2021-06-02

**Authors:** Kathryn M. Nowotny, Kapriske Seide, Lauren Brinkley-Rubinstein

**Affiliations:** 1 University of Miami Department of Sociology, 5202 University Drive, Merrick Building 120, Coral Gables, FL 33146 USA; 2 Chapel Hill Department of Social Medicine, University of North Carolina, 333 South Columbia Street, MacNider Hall, Chapel Hill, NC 27599 USA

**Keywords:** COVID-19, Occupational health, Prisons

## Abstract

**Background:**

Multiple large outbreaks of COVID-19 have been documented in prisons and jails across regions of the world, with hazardous environmental conditions amplify the risks of exposure for both incarcerated people and correctional staff. The objectives of this study are to estimate the cumulative prevalence of COVID-19 cases among U.S. prison staff over time and compare it to the prison inmate population and the general U.S. population, overall, and to examine risk of COVID-19 infection among prison staff across jurisdictions.

**Methods:**

We use publicly available data (April 22, 2020 to January 15, 2021) to estimate COVID-19 crude case rates per 1000 with 95% confidence intervals over the study period for prison staff, incarcerated population, and general population. We also compare COVID-19 case rates between prison staff and the general population within jurisdictions.

**Results:**

Over the study period, prison staff have reported consistently higher rates of COVID-19 compared to the general population, with prison staff case rates more closely mirroring the incarcerated population case rates. The rolling 7-day average case rates for prison staff, prison population, and general population on January 15, 2021 were 196.04 per 1000 (95%CI 194.81, 197.26), 219.16 (95%CI 218.45, 219.86), and 69.80 (95%CI 69.78, 69.83), respectively. There was substantial heterogeneity across jurisdictions, yet in 87% of study jurisdictions, the risk of COVID-19 was significantly greater among prison staff than the general state population.

**Conclusions:**

Targeting staff for COVID-19 mitigation strategies is essential to protect the health of people who intersect with the correctional system and to flatten the curve in the surrounding communities.

**Supplementary Information:**

The online version contains supplementary material available at 10.1186/s12889-021-11077-0.

## Background

The World Health Organization (WHO) announced on March 11, 2020, that the scale of infections caused by the novel coronavirus, COVID-19, met the threshold for a pandemic [1]. As of this writing, there are over 109 million confirmed cumulative cases of COVID-19 worldwide, with the United States accounting for 25% global cases, yet 4% of the global population [2]. Multiple large outbreaks of COVID-19 have been documented in prisons and jails across regions of the world [3], including Africa [4], Asia [5], North America, Central America, and the Caribbean [6], South America [7], and Europe [8]. Hazardous environmental conditions amplify the risks of exposure for both incarcerated people and correctional staff [9, 10]. In U.S. jails, “jail churn” of admissions and releases from local jails amplifies rates of COVID-19 transmission within these facilities and its spread to surrounding communities [11]. While people who are incarcerated suffer the most from these conditions, correctional staff--including correctional officers (COs), correctional healthcare workers, and other administrative/clerical staff--are at high risk for occupational infections [12], which can spread to their social networks outside of work.

Populations living and working in jails and prisons have historically been susceptible to infectious disease outbreaks [9], including methicillin-resistant *Staphylococcus aureus* (MRSA) [13], tuberculosis (TB) [14], influenza [15], syphilis [16], varicella-zoster virus (chickenpox) [17], and foodborne illnesses [18]. TB, for example, is highly prevalent among COs in the United States [19], and TB outbreaks in prisons have led to documented community spread [20]. Some studies have also documented a higher prevalence of TB and bloodborne exposure among correctional healthcare workers [10]. Despite this, infectious disease exposure among U.S. correctional staff remains understudied. The National Institute for Occupational Safety and Health (NIOSH) notes that COs, like most public safety workers, are regularly exposed to infectious diseases in their line of work [21], including through close contact with a population at high risk for infectious diseases [22].

In other countries, such as Greece, one study found that prison staff, along with incarcerated people, were at high risk for hepatitis B and C [23]. In Italy, the rate of hepatitis B virus is comparable between incarcerated people and prison officers [24]. In Ghana, a national multicenter cross-sectional study found a higher prevalence of human immunodeficiency virus (HIV), hepatitis B virus, hepatitis C virus, and syphilis infections among prison officers [25]. According to the WHO, TB is a major public health issue in Eastern European prison systems [26]. A systematic literature review of studies published from 1992 to 2015 on the prevalence of multidrug-resistant tuberculosis (MDR -TB) in prisons of Post-Soviet states found the prevalence to be as high as 16 times more than the worldwide prevalence estimated by the WHO [27]. These settings are reservoirs for TB outbreaks and facilitate the transmission of TB onsite and offsite through prison staff, visitors, and people who are  released from prisons. In the context of the COVID-19 pandemic, correctional staff are likely at disproportionately high risk for severe COVID-19 complications as they navigate between correctional facilities and their communities [28], and this may be compounded by other occupational risks. For example, the organizational structure and climate of correctional facilities have a consistent relationship with CO job stress and burnout [29–31]. Burnout, in turn, is related to numerous health outcomes among COs, including poor nutrition, physical inactivity, sleep duration, sleep quality, diabetes, and anxiety/depression [32], which may place people at greater risk for COVID-19 complications.

The objective of this study is to estimate the cumulative prevalence of COVID-19 cases among U.S. prison staff (e.g., COs, correctional healthcare workers, other clerical/admin) over time and compare it to the prison inmate population and the general U.S. population, overall. Previous research has estimated national prevalence for people in prison of various state and federal prison systems in the United States [33], as well as substantial variation across U.S. states [34]. Since prisons operate at different jurisdictional levels (e.g., state), we therefore examine the risk of COVID-19 infection among prison staff compared to the general population within the same jurisdiction.

## Methods

This brief report provides a descriptive analysis of publicly available data to compare the risk of COVID-19 infection among prison staff to the prison population and general population using national longitudinal data, and to compare prison staff and general population using state-level cross-sectional data. The analysis was performed using STATA 15 and Microsoft Excel. Data were publicly available and exempt from IRB review. We followed STROBE reporting guidelines.

### Data

The general population COVID-19 case data were from The New York Times as of January 15, 2021 [35], and the denominator data were from the 2019 American Community Survey [36].

Data on COVID-19 among prison staff were from the Covid Prison Project (CPP; www.covidprisonproject.com) [37] and were reported as of January 15, 2021. The Federal Bureau of Prisons, Puerto Rico, and 45 state prison systems were included in this analysis. COVID-19 case data for staff were not reported by Alaska, Connecticut, New Mexico, and North Carolina; they were removed from the analysis. Staff population data was missing for Massachusetts, and this prison system was removed from the analysis. The cases reported among prison staff are likely an undercount since most jurisdictions did not conduct testing of their employees, relying instead on self-report, with jurisdiction websites describing COVID-19 testing as the “individual responsibility of employees” [38]. Additionally, most jurisdictions did not differentiate between types of employees when reporting COVID-19 cases (e.g., CO, correctional healthcare worker, clerical, etc.).

Staff population data were collected from the department of corrections websites and other government sources. Table 1 in the supplementary file includes citations, raw numbers, and definitions from each jurisdiction included in the analysis. Departments of correction varied in the way they reported information about correctional staff, including where this content was found on their website (e.g., annual report, public data reporting), the quality of the content (e.g., explicit definitions of “staff”), and the latest year data were available (e.g., 2018 or 2020). It is also widely known that correctional settings, in general, have high staff turnover rates as well understaffing [39, 40]. Therefore, point-in-time population estimates do not fully capture the population at risk over the course of the pandemic. These points raise serious concerns about the validity of the denominator data used in this analysis. Accurate and timely data from correctional systems have been a hindrance to epidemiological investigations throughout the COVID-19 pandemic [41]. While the extant research on infectious diseases among correctional staff has been limited to COs and correctional healthcare workers, it is likely that clerical, administrative, and other staff are at increased risk of exposure as well, given their movement in and out of correctional facilities and their regular interaction with COs. Since jurisdictions do not differentiate among prison staff when reporting COVID-19 cases, we include all staff in the denominator as reported. Similar to the general population, the prison staff case rates should be considered *crude estimates* of the true case rate given these data limitations.

The population data for the incarcerated population was more valid than the staff data. We used the People in Prison data collected by the Vera Institute of Justice [42]. Prison population counts were collected during the first quarter of 2020, and largely capture the changes in population size due to COVID-19. When possible, we use the most recent count (collected on April 30th / May 1st, 2020). For states without data from this most recent wave, we use the second most recent count (December 31st, 2019 for Illinois, Maryland, Minnesota, and Virginia; and March 31st for Montana, South Dakota, Tennessee, and Washington). Data for Puerto Rico are from the Bureau of Justice Statistics and reported for 2018 [43].

### Analysis

We first examined COVID-19 crude case rates per 1000 with 95% confidence intervals over the study period for prison staff, incarcerated population, and general population. This included 269 days from April 22, 2020, to January 15, 2021. For this analysis, the prison staff and incarcerated COVID-19 and population data included the Federal Bureau of Prisons, Puerto Rico, and 45 state prison systems (excluded states were Alaska, Connecticut, New Mexico, North Carolina, and Massachusetts). The number of cases varied by day and the baseline population was 400,889 for prison staff and 1,323,766 for the incarcerated population. Daily COVID-19 cases for the general population were from the 50 U.S. states [35] and the population for the denominator was 328,239,523 [36].

Next, state-level case rates were calculated along with risk ratios. This analysis excluded the Federal Bureau of Prisons since there is not a direct jurisdictional comparison. Forty-five state prison systems and Puerto Rico were included. The COVID-19 case counts as of January 15, 2021, were from COVID Prison Project (prison staff) and The New York Times (general population). The population denominator data for prison staff are detailed in the appendix and the corresponding data for the general state populations are from 2019 American Community Survey estimates [36]. Finally, the state-level COVID-19 case rates for the general population (45 states plus Puerto Rico) were categorized into quartiles. Prison staff COVID-19 case rates were then categorized based on the general population quartile cutoffs.

## Results

**Fig. 1:**
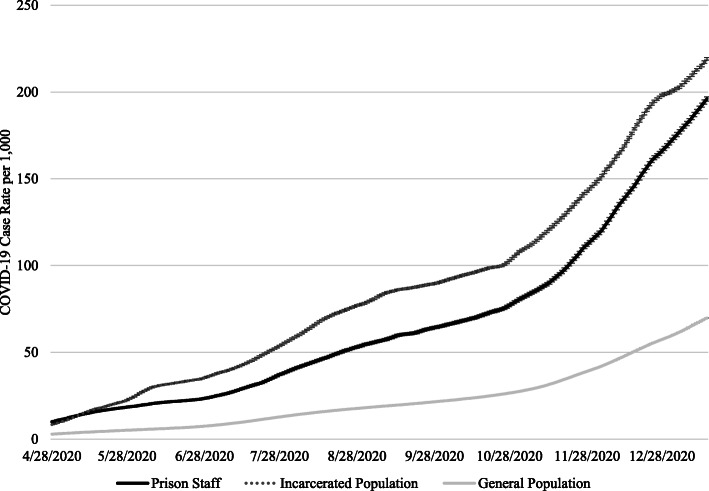
Rolling 7-Day Average COVID-19 Case Rate per 1000 with 95% Confidence Intervals for Prison Staff compare to Prison Population and General Population, April 28, 2020 to January 15, 2020

As of January 15, 2021, there have been 80,963 cases of COVID-19 reported among prison staff reported by U.S. prison systems. Over the study period, prison staff have reported consistently higher rates of COVID-19 compared to the general population (Fig. 1). For example, on April 28, 2020, the 7-day rolling average case rate for prison staff was 9.95 per 1000 (95% confidence interval (95%CI) 9.64, 10.26) and the corresponding rate for the general population was 2.85 (95%CI 2.84, 2.86). On January 15, 2021, the prison staff case rate grew to 196.04 per 1000 (95%CI 194.81, 197.26), and the general population case rate grew to 69.80 (95% CI 69.78, 69.83). Prison staff case rates more closely mirror the incarcerated population case rates, although for most of the study period prison staff case rates were lower than the prison population case rates. The 7-day rolling average case rate for the prison population on April 28, 2020, was 8.39 (95%CI 8.24, 8.55) and on January 15, 2021, it was 219.16 (95%CI 218.45, 219.86).

Table 1 compared the prison staff case rate to the general population case rate at the state-level as of January 15, 2021. Three states (Mississippi, Virginia, Wyoming) and Puerto Rico reported a lower risk of COVID-19 among prison staff than the general population. Maine reported no cases among prison staff. In 87% (40/46) of study jurisdictions (45 states and Puerto Rico), the risk of COVID-19 was significantly greater among prison staff than the general state population, with risk ratios ranging from 1.18 in Arkansas to 17.25 in Washington. The mean risk ratio was 3.04 and the median risk ratio was 2.66. The mean COVID-19 prison staff case rate was 205 per 1000 (median = 199), while the mean COVID-19 case rate in the general population of study jurisdictions was 72 per 1000 (median = 73). The correlation between the prison staff and general population case rates across jurisdictions was small (*r* = 0.29).

**Table 1:** COVID-19 Case Rates and Risk Ratios Comparing COVID-19 Case Rates among Prison Staff in State Departments of Correction Compared to the General State Population, as of January 15, 2020

	Prison Staff Case Rate per 1000 (95% Confidence Interval)	General Population Case Rate per 1000 (95% Confidence Interval)	Risk Ratio
Alabama	247 (233, 261)	85 (85, 85)	2.90
Arizona	231 (222, 239)	91 (91, 91)	2.54
Arkansas	104 (96, 113)	89 (88, 89)	1.18
California	250 (246, 253)	74 (74, 74)	3.36
Colorado	244 (233, 255)	65 (65, 65)	3.76
Delaware	293 (274, 313)	71 (70, 71)	4.16
Florida	206 (201, 211)	72 (72, 72)	2.86
Georgia	143 (136, 150)	73 (73, 73)	1.96
Hawaii	47 (37, 56)	17 (17, 17)	2.75
Idaho	180 (164, 196)	87 (86, 87)	2.08
Illinois	373 (364, 383)	84 (84, 84)	4.46
Indiana	231 (220, 241)	87 (87, 87)	2.65
Iowa	256 (239, 273)	96 (96, 97)	2.66
Kansas	312 (296, 227)	89 (89, 89)	3.50
Kentucky	259 (243, 275)	73 (72, 73)	3.57
Louisiana	200 (187, 212)	78 (78, 79)	2.55
Maine	0 (0, 0)	24 (24, 25)	–
Maryland	171 (164, 178)	53 (53, 53)	3.22
Michigan	244 (236, 251)	58 (58, 58)	4.21
Minnesota	379 (365, 393)	79 (78, 79)	4.82
Mississippi	67 (59, 76)	83 (83, 84)	0.81
Missouri	184 (176, 191)	76 (76, 76)	2.42
Montana	158 (138, 178)	83 (82, 84)	1.91
Nebraska	195 (180, 211)	94 (93, 94)	2.08
Nevada	335 (317, 353)	84 (84, 84)	4.00
New Hampshire	144 (122, 166)	41 (40, 41)	3.52
New Jersey	197 (188, 206)	69 (69, 70)	2.84
New York	197 (191, 202)	62 (62, 62)	3.15
North Dakota	313 (282, 345)	125 (125, 126)	2.50
Ohio	343 (334, 351)	70 (70, 70)	4.92
Oklahoma	188 (177, 199)	88 (88, 88)	2.41
Oregon	161 (151, 172)	31 (31, 31)	5.18
Pennsylvania	216 (209, 223)	59 (59, 60)	3.63
Puerto Rico	26 (22, 29)	37 (37, 38)	0.69
Rhode Island	196 (175, 216)	99 (98, 100)	1.98
South Carolina	168 (158, 178)	73 (73, 73)	2.30
South Dakota	241 (211, 272)	119 (118, 120)	2.03
Tennessee	266 (254, 278)	97 (97, 98)	2.74
Texas	275 (270, 280)	72 (72, 72)	3.81
Utah	133 (119, 146)	100 (100, 100)	1.33
Vermont	37 (25, 48)	16 (14, 17)	2.37
Virginia	29 (26, 32)	50 (49, 50)	0.58
Washington	658 (632, 683)	38 (38, 38)	17.25
West Virginia	105 (96, 115)	60 (59, 60)	1.77
Wisconsin	201 (193, 208)	97 (97, 97)	2.07
Wyoming	39 (28, 49)	85 (84, 85)	0.46

**Fig. 2:**
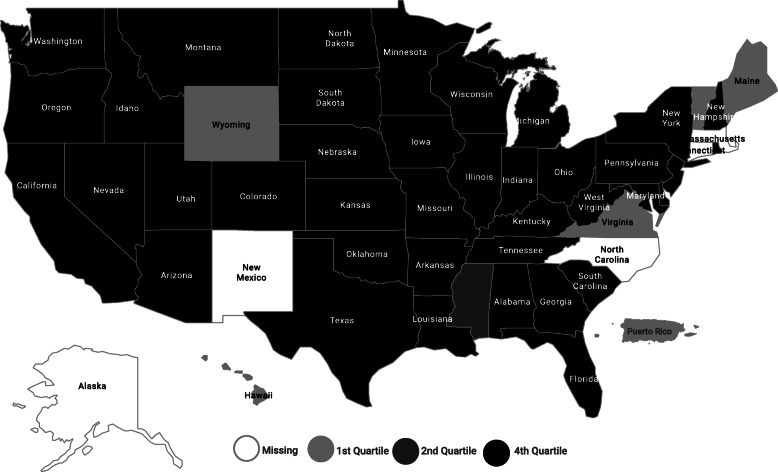
Prison Staff COVID-19 Case Rates Normed to General Population COVID-19 Case Rate Quartiles, as of January 15, 2021. Note: There are no states in the 3rd quartile. This figure was created using Infogram, a data visualization tool (www.infogram.com)

In 39 out of 46 study jurisdictions, the COVID-19 prison staff case rate was greater than 72 per 1000, the mean rate in the general population. This disparity is also demonstrated in Fig. 2. The general population case rate for the 46 study jurisdictions (45 states are Puerto Rico) were coded into quartiles: 1st quartile 0.0 per 1000 to 59.51 per 1000, 2nd quartile > 59.51 per 1000 to 75.05 per 1000, 3rd quartile > 75.05 per 1000 to 87.96 per 1000, and 4th quartile > 87.96 per 1000. We coded the prison staff case rates for the same 46 jurisdictions using these quartile cut-offs. Thirty-nine of the jurisdictional prison systems were classified in the 4th quartile (> 87.96 per 1000) based on general population rates of study jurisdictions, compared to 11 out of 46 in the general population.

## Discussion

The cumulative case rate for COVID-19 among prison staff outpaces the general population and more closely mirrors the prison population. We found considerable heterogeneity in relative risk for COVID-19 among prison staff compared to the general population across study jurisdictions. Nevertheless, in 87% of study jurisdictions the risk of COVID-19 infection was greater among prison staff than the general population.

Several limitations need to be considered when interpreting these findings. First, as previously mentioned, the COVID-19 cases reported among prison staff are most likely an undercount, which means that our estimates are conservative. Second, the prison staff population data is problematic due to the reasons outlined in the Methods section of this paper. It is possible that for some states the population denominator is inflated leading to an underestimation of the cumulative case rate, while for other jurisdictions the denominator is deflated leading to an overestimation of the cumulative case rate. The point-in-time estimates also do not accurately capture the total population at-risk given the high turnover rates among people who work in prisons. In the absence of mandates for timely reporting of high-quality data, we are left with the data that is made available by jurisdictions [41]. Third, this study did not adjust for demographic and other population differences across groups. Like the incarcerated population, Black people are overrepresented among COs and jailers, accounting for 11.6% of the U.S. workforce and 23.8% of COs and jailers [44]. In the Federal Bureau of Prisons, African Americans account for 21.3% of all staff [45]. Given how structural racism drives inequity across the COVID-19 continuum [46–48], adjusting for racial composition, for example, may be necessary for understanding differential risks for COVID-19 among correctional and non-correctional populations [49].

In April 2020, Ahalt [50] warned that, “Incarcerated people, corrections officers, and their families and communities are bound together by the threat of a deadly and fast-moving disease [COVID-19]. The sooner we recognize this, and take decisive action, the more lives we will save.” Our analysis supports this warning: correctional staff have a “shared fate” with incarcerated people. The cumulative case rate for COVID-19 among prison staff was lower than for people in prison but has trended upward at similar rates. The heterogeneity among COVID-19 case rates among prison staff is likely due to varying state policies [38]. There is substantial variation in COVID-19 communication policies, quarantine and isolation policies, and resident and staff masking policies. For example, as of October 2020 only 68% of U.S. prison jurisdictions reported having a policy requiring staff to wear masks [51]. However, given the decentralized response to the COVID-19 pandemic in the United States, there is a variety of factors that could explain variation across jurisdictions [52].

## Conclusions

The COVID-19 pandemic has exposed the need for progressive criminal justice reforms [53], including decarceration [54]. It has also laid bare the necessity of protecting the health and safety of correctional workers as a moral imperative [55]. As noted by others [55], occupational health interventions should focus primarily on COs given their high proportion among correctional staff and close contact with incarcerated people; however, COVID-19 mitigation interventions should be adapted to reduce risk for infection for all staff.

## Supplementary Information

**Additional file 1.**

## Data Availability

The data used in this study are publicly available from 51 different sources and are available from the corresponding author on reasonable request. Detailed references including weblinks are provided for all 51 data sources in the References and the Supplementary Table.

## Abbreviations

CPP: Covid Prison Project
CO: Correctional officer
HIV: Human Immunodeficiency Virus
NIOSH: National Institute for Occupational Safety and Health
MRSA: Methicillin-resistant *Staphylococcus aureus*
MDR - TB: Multidrug-resistant Tuberculosis
TB: Tuberculosis
WHO: World Health Organization
