# Supplementary material for: Risk of COVID-19 infection among prison staff in the United States
Source: BMC Public Health. 2021 Jun 2;21:1036. doi: 10.1186/s12889-021-11077-0 (PMC8170443; doi:10.1186/s12889-021-11077-0)
Supplement: Supplementary file 1 — Additional file 1. [file 12889_2021_11077_MOESM1_ESM.docx]

| Supplementary Table 1. Staff Population Data, Definitions, and Sources | | | | |
| --- | --- | --- | --- | --- |
|  | Staff | Reporting  Year | Definition | Source |
| Alabama | 3,496 | 2019 | Defined as budgeted positions. | State of Alabama. (2020). *Executive Budget Fiscal Year 2020*. Retrieved from https://budget.alabama.gov/wp-content/uploads/2019/04/FY-2020-BUDGET-DOCUMENT.pdf |
| Arizona | 9,556 | 2019 | Defined as full-time equivalent (FTEs). | Arizona State Legislature – The Joint Legislative Budget Committee. (2020). *FY 2021 Baseline Book.* Retrieved from https://www.azleg.gov/jlbc/21baseline/adc.pdf (pp. 110 – 119). |
| Arkansas | 4,619 | 2019 | 4,619 Total: 3,502 "security personnel," 1,117 "non-security" | Arkansas Division of Correction (ADC) – The ADC Research and Planning Division. (2019). *Annual Report Fiscal Year 2019.* Retrieved from https://adc.arkansas.gov/images/uploads/Division_of_Correction_FY19_Annual_Report_BOC_Approval-5272020.pdf (pp. 6) |
| California | 57,140 | 2018 | Defined as "authorized positions." | State of California. (2020). *Governor’s Budget Summary 2020-21*. Retrieved from http://www.ebudget.ca.gov/2020-21/pdf/BudgetSummary/FullBudgetSummary.pdf (pp. 261) |
| Colorado | 6,083 | 2018 | No definition listed. | State of Colorado - Joint Budget Committee. (2020). *Staff Budget Briefing FY 2020-21 Department of Corrections.* Retrieved from http://leg.colorado.gov/sites/default/files/fy2020-21_corbrf.pdf |
| Delaware | 2,110 | 2019 | 2,100 Total: 1,753 corrections officers, 357 "support staff." | Delaware Department of Correction Annual Report. (2019). Retrieved from https://doc.delaware.gov/assets/documents/annual_report/DOC_2019AnnualReport.pdf (pp.11) |
| Florida | 24,000 | 2020 | This is "approximate," states that the "majority" are correctional officers. | Florida Department of Corrections – Office of Inspector General. (2020) *Annual Report Fiscal 2018-19.* http://www.dc.state.fl.us/ig/igannual/OIG_Annual_Report_FY2018-19%20FINAL.pdf (pp. 8) |
| Georgia | 9,169 | 2019 | 50.9% COs, 91.6% in "Facilities Division" "providing direct supervision of the offenders," 16.7% "sworn employees." | Georgia Department of State. (2019). *Fiscal Year 2019*. Retrieved from http://www.dcor.state.ga.us/sites/default/files/sites/all/gdc/files/pdf/Research/Monthly/GDC%20FY2019%20Annual%20Report.pdf (pp. 14) |
| Hawaii | 1,909 | 2020 | Staff who work in correctional facilities. | personal communication |
| Idaho | 2,200 | 2019 | No definition listed. | White, Janeena. (2019). *Idaho Department of Correction. Idaho Prison*  *Compstat* presented at the 7th Annual ICRN/NCRP meeting. Retrieved from https://www.cjinstitute.org/assets/sites/2/2019/11/09.19-1300-Plenary-Research-in-Corrections-Practices-Janeena-White-Idaho-Prison-Compstat-Reporting.pdf |
| Illinois | 9,852 | 2019 | 9,852 Total: 8,792 "Security," 2,772  "Non-Security." Total does not include 1,712 "Non-DOC" staff. | Illinois Department of Corrections. (2019). *Operations and Management Report (OMR) Key Variables Fiscal Year 2019*. Retrieved from https://www2.illinois.gov/idoc/reportsandstatistics/Documents/JHA%20JUN%20FY2019.pdf |
| Indiana | 6,473 | 2019 | 6,473 Total: "Total Department Active Personnel," includes 532 "GEO Contracted Non-State Employees." | Indiana Department of Corrections. (2019). *Fiscal Year 2019 Annual Report*. Retrieved from  https://www.in.gov/idoc/files/2019%20DOC%20Annual%20Report%206.29.20.pdf |
| Kansas | 3,562 | 2019 | 3,562 Total: 2,217 "Uniformed," 1,345 "Non-Uniformed." | Kansas Department of Corrections. (2019). *Fiscal Year 2019 Annual* https://www.doc.ks.gov/publications/Reports/fy-2019-kdoc-annual-report. (pp. 26) |
| Kentucky | 2,880 | 2018 | From Annual Report, added up all facility staff totals. | Kentucky Department of Corrections. (2018). *2018 Annual Report.* https://corrections.ky.gov/About/researchandstats/Documents/Annual%20Reports/2018%20Annual%20Report.pdf |
| Louisiana | 3,973 | 2019 | 3,973 Total: 65 "Prison Enterprises,"  3,656 "Institutions," 252 "Headquarters." | Louisiana Department of Public Safety and Corrections. (2019). *2019 Annual Report*. Retrieved from https://s32082.pcdn.co/wp-content/uploads/2020/01/Fiscal-Year-2019-Annual-Report-FINAL-1.17.20.pdf (pp. 8) |
| Maine | 2,918 | 2018 | Defined as full-time equivalent (FTEs). | Maine State Legislature  Office of Fiscal and Program Review. (2018). *General Fund Appropriations*  *2018-2019 Biennium*  *Through 128th Legislature, 2nd Special* *Session*. Retrieved from http://legislature.maine.gov/doc/2551 |
| Maryland | 10,412 | 2020 | Defined as full-time equivalent (FTEs). | Maryland Department of Budget and Management. (2020). *FY 2020 Proposed Operating Budget Detail by Agency*. Retrieved from https://dbm.maryland.gov/budget/Pages/operbudget/fy2020-agency.aspx |
| Michigan | 12,145 | 2018 | 12,415 Total: 5,927 are COs, Corrections Medical Officers (CMOs) and Corrections Medical Unit Officers (CMUOs). | Michigan Department of Corrections. (2019). *2018 Statistical Report.* Retrieved from https:/[/www](http://www.michigan.gov/documents/c).[michigan.gov/documents/c](http://www.michigan.gov/documents/c) orrections/MDOC_2018_Statistical_Rep ort_-_2019.07.18_662129_7.pdf (pp. F–19) |
| Minnesota | 4,379 | 2018 | No definition listed. | Minnesota Department of Corrections. (2018). Retrieved from https://mn.gov/doc/assets/2018%20DOC%20Performance%20Report_tcm1089-366101.pdf  -366101.pdf (pp. 31) |
| Mississippi | 3,270 | 2019 | 3270 total: 1872 authorized FTE in state prison systems; 987 security and 411 non-security staff in private and regional correctional facilities | Mississippi Department of Corrections. (2019). *FY 2019 Annual Report*. Retrieved from https://www.mdoc.ms.gov/Admin-Finance/Documents/2019%20Annual%20Report.pdf (PP. 41-43) |
| Missouri | 11,233 | 2019 | Defined as full-time equivalent (FTEs). | Missouri Office of the Governor. (2020). *The Missouri Budget. Fiscal Year 2020*. Retrieved from https://oa.mo.gov/sites/default/files/FY_2020_Executive_Budget_Final.pdf (pp. 9-1) |
| Montana | 1,288 | 2020 | Defined as full-time equivalent (FTEs). | State of Montana Governor’s Office of Budget and Program Planning. (2020). Section D: Judicial Branch, Law Enforcement, & Justice. Retrieved from http://budget.mt.gov/Portals/29/execbudgets/2021_budget/Executive%20Budget/Section%20D%20Final.pdf?ver=2018-11-15-095921-397 (pp. D-47) |
| Nebraska | 2,507 | 2020 | 2,507 total: 1,380.50 protective services,  70.50 nurses, 27.50 education, 1,028.50 other | Nebraska Department of Correctional Services. (2020). *NDCS Quarterly Population Summary January - March 2020*. Retrieved from https://corrections.nebraska.gov/sites/default/files/ndcs_quarterly_data_sheet_fy20-q3_0.pdf (pp. 8) |
| Nevada | 2,627 | 2020 | Defined as full-time equivalent (FTEs). | Nevada Department of Corrections. (2020). *Stat Facts Fiscal Year 2021*. Retrieved from http://doc.nv.gov/uploadedFiles/docnvgov/content/About/Statistics/WFS/20200823.Factsheet.pdf |
| New Hampshire | 974 | 2018 | 974 total: 12 executives, 27  managers/administrators, 515 correction officers, 72 probation/parole officers, 65 medical personnel, 29 teachers/instructors, 69 shop supervisors/tradesmen, 3 recreation personnel, 115 clerical/administrative, 38 correctional counselor/case managers, 29 psychological/social workers | New Hampshire Department of Corrections. (2018). *SFY2018 Annual Report*. Retrieved from https://www.nh.gov/nhdoc/divisions/publicinformation/documents/nhdoc-annual-report-2018-final-hhbrj.pdf (pp. 55) |
| New Jersey | 8,000 | 2020 | No definition listed. This approximates "nearly 8000 staff." | State of New Jersey Office of Management and Budget. (2020). *The Governor’s FY2021 Budget - Detailed Budget*. Retrieved from https://www.nj.gov/treasury/omb/publications/21budget/pdf/FY21GBM.pdf (pp. D-65) |
| New York | 19,094 | 2018 | 19,094 total: corrections officers, sergeants and lieutenants. Total does not include non-security staff. | The Assembly State of New York Albany – Standing Committee on Correction. (2018). *2018 Annual Report*. Retrieved from https://nyassembly.gov/write/upload/postings/2019/pdfs/20190319_0085751.pdf (pp. 5) |
| North Dakota | 836 | 2017 | 836 total includes 323 Correctional Officer I, Correctional Supervisor II or Captain, 36 Juvenile Institutional Residence Specialists and Security Officers, 67 direct care medical and treatment and an additional 90 FTE responsible for education and case management services, 100 temporary positions. | North Dakota Department of Corrections and Rehabilitation. (2017). 2015-2017 Biennial Report. Retrieved from https://www.docr.nd.gov/sites/www/files/documents/Biennial%20Report%20Archive/Biannual%20Report%202015-2017.pdf (pp. 14) |
| Ohio | 12,267 | 2020 | Includes 6,709 total correction officers and 511 parole officers. The rest of the staffing profile is not included in this report. | Ohio Department of Rehabilitation and Correction. (2020). J*uly Fact Sheet - Staff Profile Section*. Retrieved from https://drc.ohio.gov/Portals/0/July%20fact%20sheet.pdf |
| Oklahoma | 4,725 | 2019 | defined as FTE; no breakdown provided. | State of Oklahoma. (2020). FY2021 Executive Budget. Retrieved from https://omes.ok.gov/sites/g/files/gmc316/f/publications/bud21.pdf (pp. 176) |
| Oregon | 4,579 | 2018 | 4,579 total: 2,572 security staff, 1,861 non-security staff, 41 parole and probation officers and 105 temporary employees (found 2596 security; 1889 non-security; 36 parole, 92 temps for November 2020) | Oregon Department of State. (2019). Issue Briefing. Retrieved from https://www.oregon.gov/doc/Documents/agency-quick-facts.pdf |
| Pennsylvania | 14,538 | 2018 | 14,538 totals: 9,372 security staff and 5,166 other staff positions | Pennsylvania Department of Corrections. (2018). *Annual Report 2018*. Retrieved from https://www.cor.pa.gov/About%20Us/Statistics/Documents/Reports/2018%20Annual%20Statistical%20Report.pdf (pp. 31) |
| Puerto Rico | 7,809 | 2019 | Defined as full-time equivalent (FTEs). | Financial Oversight and Management Board for Puerto Rico. (2019). *2019 Fiscal Plan for Puerto Rico Restoring Growth and Prosperity*. Retrieved from https://cases.primeclerk.com/puertorico/Home-DownloadPDF?id1=OTEyNDY1&id2=0 |
| Rhode Island | 1,411 | 2019 | 1411 total FTE: 1384 classified, 27  unclassified | State of Rhode Island and Providence Plantations. (2021). Budget Volume IV – Public Safety, Natural Resources and Transportation: Department of Corrections. Retrieved from http://www.omb.ri.gov/documents/Prior%20Year%20Budgets/Operating%20Budget%202021/BudgetVolumeIV/2_Department%20of%20Corrections.pdf |
| South Carolina | 5,010 | 2018 | 5,010 total: 2,906 security, 2,104 non- security | South Carolina Department of Corrections. (2018). *SCDC Staff Report Security vs Non-Security Filled Positions as of October1, 2018*. Retrieved from https://www.scstatehouse.gov/CommitteeInfo/Ways&MeansMeetingHandouts/Law%20Enforcement/SCDC%20FY20%20Budget%20Hearing.pdf |
| South Dakota | 750 | 2018 | 750 total (positions unknown): 4 at STAR Academy, 22 Administration, 16 Pheasantland Industries, 27 Inmate Services, 35 Juvenile Community Corrections, 57 Parole, 70 Women's prison, 210 Mike Durfee State Prison, 309 State Penitentiary | South Dakota Department of Corrections. (2018). *FY 2018 Annual Report*. Retrieved from https://doc.sd.gov/documents/about/publications/SDDOCFY2018AnnualReportFINAL.pdf (pp. 26) |
| Tennessee | 5,245 | 2019 | 5,245 total: 3,382 security and 1,839 non- security/administrative | Tennessee Department of Correction. (2019). *Annual Reports - Annual Statistical Abstracts, Fiscal Year 2019*. Retrieved from https://www.tn.gov/content/dam/tn/correction/documents/StatisticalAbstract2019.pdf (pp. 7) |
| Texas | 29,678 | 2018 | 29,678 total: 25,491 correctional officers and ranking officers (sergeant through major), 4,187 staff members who worked as wardens, operational, technical and unit support staff | Texas Department of Criminal Justice. (2018). Annual Review 2018. Retrieved from https://www.tdcj.texas.gov/documents/Annual_Review_2018.pdf (p. 18) |
| Utah | 2,446 | 2019 | defined as FTE *Note: These are allotted positions from a budget report. | Utah Office of the Legislative Fiscal Analyst. (2019). *Budget of the State of Utah and Related Appropriations 2019-2020.* Retrieved from https://le.utah.gov/interim/2019/pdf/00002717.pdf (pp. 3-27) |
| Vermont | 1,028 | 2019 | 1,028 "total authorized positions" Note: These are allotted positions from a budget report. | Vermont Department of Corrections. (2020). FY 2020 Budget Presentation. https://doc.vermont.gov/sites/correct/files/documents/reports/doc-fy20-budget-presentation.pdf (pp. 15) |
| Virginia | 11,363 | 2019 | Defined as "average employment level." | Virginia Department of Corrections. (2019). *Management Information Summary Annual Report for the Fiscal Year Ending June 30, 2019*. Retrieved from https://vadoc.virginia.gov/media/1466/vadoc-financial-annual-mis-report-2019.pdf (pp. 32) |
| Washington | 1,350 | 2020 | 1,350 total: 82.2% security and control, 8.0% administration, 5.1% Programs and case management, 1.9% Director's office, 1.7% Community corrections, 1.1% Agency fiscal office | District of Columbia Department of Corrections. (2020*). Fact and Figures April 2020*. Retrieved from https://doc.dc.gov/sites/default/files/dc/sites/doc/publication/attachments/DCDepartmentofCorrections_FactsandFigures_April2020_0.pdf |
| West Virginia | 4067 | 2020 | Defined as "total budgeted FTE positions" | State of West Virginia. (2021). Executive Budget: Volume I Budget Report Fiscal Year 2021. Retrieved from https://budget.wv.gov/executivebudget/Documents/FY%202021%20Volume%20I%20Budget%20Report.pdf (pp. 59) |
| Wisconsin | 10,436 | 2020 | Defined as full-time equivalent (FTEs). | State of Wisconsin Department of Corrections. (2018). Agency Budget Request 2019 – 2021 Biennium. Retrieved from https://doa.wi.gov/budget/SBO/2019-21%20410%20DOC%20Budget%20Request.pdf |
| Wyoming | 1,240 | 2018 | 1,240 total: 1,226 FTEs, 3 part-time employees, 11 AWECs. The staffing total does not include contracted service providers such as medical and mental  health staff, and substance abuse | Wyoming Department of Corrections. (2018). *Annual Report*. Retrieved from https://docs.google.com/a/wyo.gov/viewer?a=v&pid=sites&srcid=d3lvLmdvdnxkb2N8Z3g6MTgwYjY2OGUwMWI4YjU1Yw |
